# Advanced Neuroimaging Approaches to Pediatric Brain Tumors

**DOI:** 10.3390/cancers14143401

**Published:** 2022-07-13

**Authors:** Rahul M. Nikam, Xuyi Yue, Gurcharanjeet Kaur, Vinay Kandula, Abdulhafeez Khair, Heidi H. Kecskemethy, Lauren W. Averill, Sigrid A. Langhans

**Affiliations:** 1 Department of Radiology, Nemours Children’s Hospital, Delaware, Wilmington, DE 19803, USA; xuyi.yue@nemours.org (X.Y.); vinay.kandula@nemours.org (V.K.); abdulhafeez.khair@gmail.com (A.K.); heidi.kecskemethy@nemours.org (H.H.K.); lauren.averill@nemours.org (L.W.A.); 2 Nemours Diagnostic & Research PET/MR Center, Nemours Children’s Hospital, Delaware, Wilmington, DE 19803, USA; 3 Department of Neurology, Nemours Children’s Hospital, Delaware, Wilmington, DE 19803, USA; gk2626@cumc.columbia.edu; 4 Nemours Biomedical Research, Nemours Children’s Hospital, Delaware, Wilmington, DE 19803, USA

**Keywords:** pediatrics, brain tumor, positron emission tomography, volumetrics, elastography

## Abstract

**Simple Summary:**

After leukemias, brain tumors are the most common cancers in children, and early, accurate diagnosis is critical to improve patient outcomes. Beyond the conventional imaging methods of computed tomography (CT) and magnetic resonance imaging (MRI), advanced neuroimaging techniques capable of both structural and functional imaging are moving to the forefront to improve the early detection and differential diagnosis of tumors of the central nervous system. Here, we review recent developments in neuroimaging techniques for pediatric brain tumors.

**Abstract:**

Central nervous system tumors are the most common pediatric solid tumors; they are also the most lethal. Unlike adults, childhood brain tumors are mostly primary in origin and differ in type, location and molecular signature. Tumor characteristics (incidence, location, and type) vary with age. Children present with a variety of symptoms, making early accurate diagnosis challenging. Neuroimaging is key in the initial diagnosis and monitoring of pediatric brain tumors. Conventional anatomic imaging approaches (computed tomography (CT) and magnetic resonance imaging (MRI)) are useful for tumor detection but have limited utility differentiating tumor types and grades. Advanced MRI techniques (diffusion-weighed imaging, diffusion tensor imaging, functional MRI, arterial spin labeling perfusion imaging, MR spectroscopy, and MR elastography) provide additional and improved structural and functional information. Combined with positron emission tomography (PET) and single-photon emission CT (SPECT), advanced techniques provide functional information on tumor metabolism and physiology through the use of radiotracer probes. Radiomics and radiogenomics offer promising insight into the prediction of tumor subtype, post-treatment response to treatment, and prognostication. In this paper, a brief review of pediatric brain cancers, by type, is provided with a comprehensive description of advanced imaging techniques including clinical applications that are currently utilized for the assessment and evaluation of pediatric brain tumors.

## 1. Introduction 

The most prevalent types of cancer in children in the United States (US) are leukemias, brain and central nervous system (CNS) tumors, and lymphomas. The occurrence of types of cancer varies by age and other factors. In the 0–14 years age group, leukemias, brain and other CNS tumors, lymphomas, neuroblastoma, kidney tumors, and malignant bone tumors are most common, while in adolescent children (aged 15 to 19 years), brain and other CNS tumors and lymphomas most frequently occur, followed by leukemias and other cancers [1]. The survivability of pediatric cancer has improved in recent decades due to treatments for leukemia, but now brain cancer is the leading cause of death in children [2]. Pediatric gliomas are the most common brain tumors in children [3]. Embryonal tumors and pilocytic astrocytomas (PAs) are the most prevalent tumors before 9 years of age, and gliomas grade II to Ill are most predominant until 19 years of age. The overall survival (OS) of children with brain tumors is 70% at 10 years, but this figure includes benign tumors for which complete resection is possible. OS is very low for some pediatric high-grade gliomas, such as diffuse intrinsic pontine glioma (<10–15% survival at 5 years; mean survival, 11 months), while survival is higher in patients with medulloblastoma (all subgroups combined, 53% at 10 years) [4]. In contrast, OS in children with low-grade gliomas is 85% to 90%, including cases in which the tumor cannot be resected [4].

Neuroimaging plays an invaluable role in the diagnosis, treatment, and monitoring of pediatric brain tumors. While traditional imaging techniques are good at detecting pediatric brain cancer, they offer low specificity, lack of histological correlation [5], and have limited capacity to evaluate therapeutic response evaluation. New targeted chemotherapy and radiation therapies result in imaging changes, indicating either pseudo-response or pseudo-progression, which are difficult to adequately assess with conventional morphologic imaging techniques [6]. The incorporation of various advanced neuroimaging strategies, such as magnetic resonance spectroscopy (MRS) and perfusion-weighted imaging (PWI) has led to improved diagnostic accuracy in differentiating tumor recurrence from treatment-induced changes [7,8,9]. Although studies utilizing positron emission tomography (PET) with ^18^F- and ^11^C-labelled radiotracers and FDG (2-Deoxy-2-[^18^F]fluoro-D-glucose) have demonstrated up to 96% accuracy in identifying pseudo-progression in high-grade gliomas [9], head-to-head studies comparing nuclear medicine and advanced neuroimaging techniques are not yet available [10]. 

Advanced imaging techniques, however, provide valuable insight about pediatric brain tumors by offering the quantitative and qualitative assessment of brain microstructures and physiological changes due to pathology. Furthermore, advanced imaging techniques allow for non-invasive “virtual histology” capabilities, the evaluation of function, and the assessment of tissue properties. They also provide necessary information for surgical planning, the monitoring of treatment, and the evaluation of changes in tumor status.

In this paper, a brief review of pediatric brain cancers, by type, is provided followed by a comprehensive description of advanced imaging techniques. These techniques include both those with clinical applications that are currently utilized for the assessment and evaluation of pediatric brain tumors and imaging modalities that are still being assessed for their broader value in the clinic.

## 2. Pediatric Brain Tumors

### 2.1. Overview of Pediatric Brain Tumors 

Pediatric brain tumors are the most common solid tumors in children. Traditionally classified by location, age at presentation, and histological type, advances in molecular biology and genetics have allowed for more refined subgrouping within major tumor types. Given the major implications of molecular subgrouping for diagnosis, prognosis, and treatment, in 2016, the World Health Organization (WHO) introduced a new framework for the classification of CNS tumors by integrating molecular and genetic profiling into diagnosis (Table 1) [11,12,13,14,15]. The fifth edition of the WHO classification of *Tumors of the Central Nervous System* was recently published in 2021 and represents the sixth version of the international standard for the classification of brain and spinal cord tumors [16]. Similarly, the increasing ability of radiogenomics to correlate imaging features with underlying disease biology and the advent of deep machine learning are expected to significantly contribute to improving the accuracy of diagnosis, the prediction of disease outcomes, and therapeutic decision-making for children diagnosed with brain tumors.

### 2.2. Medulloblastoma

**Figure 1:**
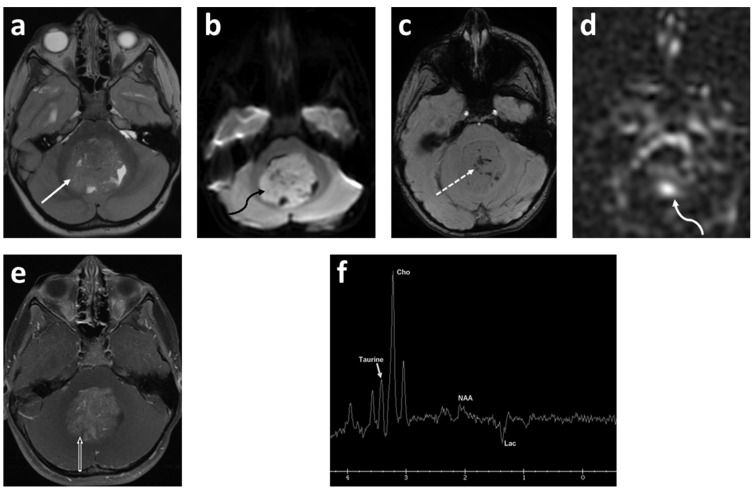
Five-year-old female presented with headache, vomiting and gait disturbance. Axial T2 (**a**); axial DWI (**b**); axial SWAN (**c**); axial ASL-PWI (**d**); axial T1 fat saturated post contrast (**e**) images; and long TE (144 msec) spectroscopy (**f**). There is a large heterogeneous, intermediate T2 signal mass centered in the fourth ventricle (solid white arrow). This mass demonstrates restricted diffusion (curved black arrow) with numerous punctate foci of the hemorrhage (dashed arrow) and increased perfusion (curved white arrow) in the center of the lesion. There is moderate heterogeneous enhancement after contrast ministration (open arrow). Spectroscopy demonstrates Taurine peak at 3.4 ppm, high choline (Cho) and undetectable N-acetylaspartate (NAA). There is also high lactate (Lac) demonstrated as an inverted peak on this long TE spectroscopy. Final diagnosis was medulloblastoma, Group 3 (author’s institutional human ethics committee/institutional review board guidelines were followed for anonymized images).

Medulloblastoma, the most predominant malignant brain tumor in children, has been at the forefront of the molecular subgrouping of pediatric brain tumors (Figure 1). Originating from precursor cells in the cerebellum or dorsal brainstem, medulloblastoma is an embryonal tumor located in the posterior fossa and comprises up to 20% of all pediatric brain tumors [17]. Previously classified into four different histological groups (classic, desmoplastic/nodular, extensive nodularity, and large cell/anaplastic tumors), medulloblastoma has now been reclassified into four groups incorporating both histology and molecular profiling. The present four distinct subgroups—wingless/integrated (WNT), sonic hedgehog (SHH), Group 3, and Group 4—have different underlying genetic alterations, distinct tumor biology, a diverse and wide range of phenotypes, and different patient outcomes [18]. Radiomic and machine learning approaches are currently being developed to predict the molecular subgroups of medulloblastoma by imaging [19].

Tumors of the WNT subgroup occur mostly in older children, account for about 10% of medulloblastomas, are almost always of classic histology, and have the most favorable prognosis among all medulloblastoma subgroups. Named after the aberrant activation of the WNT signaling pathway caused by mutations of the *CTNNB1* gene that encodes β-catenin, WNT tumors are also reported to have monosomy chromosome 6, usually in conjunction with *CTNNB1* mutations and mutations in *DDX3X, SMARCA4*, and *TP53* [12,13,14,15,18]. WNT medulloblastoma is thought to arise outside the cerebellum from cells of the dorsal brainstem, including the lower and upper rhombic lip [20]. Owing to this cellular origin, WNT medulloblastomas are typically located in the posterior fossa with approximately 75% of tumors occurring along the cerebellar peduncle and the cerebellopontine angle cistern, yielding an imaging finding that is predictive of this subgroup [21].

Comprising about 30% of medulloblastomas, SHH-driven tumors are the second most common subgroup. Mutations of signaling molecules within the SHH pathway, such as SUFU, smoothened (SMO), PTCH1, GLI1 and GLI2, are defining, and they are commonly sought after as prime targets for small molecule inhibitors in medulloblastoma drug discovery [12,13,14,15,18,22]. However, SHH tumors are indeed a highly heterogenous subgroup with varied histology ranging from classic to large cell/anaplastic and SHH subtype-specific desmoplastic/nodular medulloblastomas with extensive nodularity (MBEN). Like WNT tumors, the SHH subgroup often presents with additional mutations such as MYCN and TP53 that are defining for prognosis [23]. Because of this heterogeneity, the varying propensity to metastasize, and the varied outcomes that are also dependent on age, SHH medulloblastoma may be better defined by the following subtypes: SHHα, typically found in children with TP53 mutations; SHHβ, found in infants with poor prognosis; Shhγ, found in infants with good prognosis; and SHHΔ (or alternatively SHH1-4), tumors mostly found in adults [18,24,25] (Table 1) [16]. Originating from precursor cells of cerebellar granule neurons, SHH medulloblastomas are usually located in the cerebellar hemispheres but radiologically can also be found in the midline and then are essentially indistinguishable from Group 3 and Group 4 tumors [24].

Group 3 and Group 4 tumors are more heterogenous than WNT and SHH tumors and are still less well understood in terms of underlying genetic mutations and cells of origin. Group 3 medulloblastoma accounts for about 25% of medulloblastoma tumors and is a highly aggressive form with a propensity to metastasize. MYC amplification is frequently found in Group 3 medulloblastoma and is associated with an overall poor prognosis, but other pathways such as TNF (tumor necrosis factor)-β signaling can also be affected in tumors of this subgroup [13,14,15]. Group 4 medulloblastoma accounts for approximately 35% of tumors and, like Group 3, is biologically poorly characterized. The loss of chromosomes 8, 11 and 17p, or the gain of chromosomes 7 and 17q have been found, in addition to the amplification of CDK6, MYCN and SNCAP1 and aberrant ERBB4-SRC and nuclear factor kappa B (NF-κB) signaling [12,13,14,15,18,26,27]. Following the lead of the WNT and SHH subgroups in molecular and biological stratification, an international meta-analysis study has recently suggested that Group 3/4 may be further divided into eight subtypes (types 1–8) based on transcriptomes, DNA methylation profiling, and clinico-pathological features [16,28]. Because the radiographic features of Group 3 and Group 4 medulloblastomas are similar with midline masses arising from the vermis, advanced imaging methods are the most promising in delineating subgroup and subtype-specific characteristics for a radiological differential diagnosis of these tumors (Table 2).

### 2.3. Glioma

Gliomas are a group of highly heterogenous tumors that represent 47% of all pediatric brain tumor cases. Depending on the glial cell type of origin, gliomas are categorized into astrocytomas, ependymomas, oligodendrogliomas or astrocytic gliomas, and can range from rather benign low-grade gliomas (LGGs) to highly malignant high-grade tumors (HGGs) [14,16,17,31,32].

LGGs are the most common gliomas and are typically associated with the aberrant activation of the Ras-mitogen-activated protein kinase (MAPK) signaling pathway. They tend to be slow-growing, benign tumors, and in children malignant transformation is uncommon. LGGs encompass a variety of histologically diverse neoplasms and include pilocytic astrocytoma (grade I), subependymal giant cell astrocytoma (grade I), IDH mutant diffuse astrocytoma (grade II), IDH mutant or 1p/19q deletion oligodendroglioma (grade II), pleomorphic xanthoastrocytoma (grade II), angiocentric glioma (grade I), choroid glioma of the third ventricle (grade I or II), gangliocytoma (grade I), ganglioglioma (grade I or II), desmoplastic infantile astrocytoma, and ganglioglioma (grade I) [33,34]. Mutations usually occur in the serine/threonine kinase B-Raf proto-oncogene (BRAF) within the RAS/MAPK pathway, and specific mutations can be associated with a certain tumor type. For example, KIAA1549:BRAF fusion is mostly found in pilocytic astrocytoma and the BRAF V600E mutation is frequently detected in pilomyxoid astrocytoma and gangliogliomas [35]. Other mutations such as kRAS, FGFR1, MYB/MYBL1, NTRK2, NF1, TSC1/2 and other genetic alteration have also been identified but, unlike in adults, IDH mutations are almost absent in pediatric LGGs [12,13,14,15,32,36,37]. Interestingly, recent studies have discovered some overlap in molecular profiling between LGG and HGG, and BRAF V600E and FGFR1 mutations can be found both in LGG and HGG [32,36], suggesting that LGG and HGG might share a similar biological mechanism of tumor pathogenesis. While LGGs are heterogenous, the spatial clustering of individual tumor phenotypes and the spatial enrichment of specific genetic mutations highlight the importance and potential of the radiohistogenomic profiling of LGGs [38]. 

**Figure 2:**
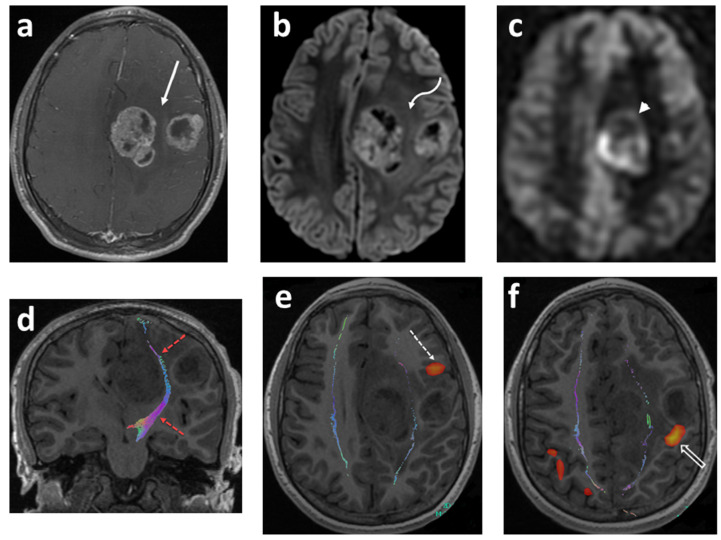
Fourteen-year-old boy with headaches and episodes of right facial and hand numbness. Axial T1 fat sat post contrast (**a**); axial DWI (**b**); axial ASL-PWI (**c**); coronal T1 tractography (**d**); and functional (**e**,**f**) images. There are two well-defined lobulated heterogeneously enhancing lesions within the posterior aspect of the left frontal lobe (solid white arrow). The lesions show restricted diffusion (curved white arrow) and increased perfusion (arrowhead). The cortical spinal tracts are identified tracking in between the two tumor masses (red–dashed arrows). The Broca’s area is identified in the left inferior frontal gyrus anterior and inferior to the lateral frontal mass lesion (dashed arrow). The motor cortex with right finger tapping is immediately posterior to the smaller mass in the lateral aspect of the left frontal lobe (open arrow). Pathology: giant cell glioblastoma (author’s institutional human ethics committee/institutional review board guidelines were followed for anonymized images).

HGGs are relatively uncommon pediatric gliomas (comprising 3–7% of all pediatric brain tumors), but they are highly aggressive and diffusely infiltrate malignant tumors yielding a very poor prognosis. They pose a serious challenge in pediatric oncology. Based on histological and radiological features, HGGs are subclassified into the following groups: anaplastic astrocytoma (grade III), IDH wild-type glioblastoma (grade IV), IDH-mutant glioblastoma (grade IV), H3K27M-mutant diffuse midline glioma (grade IV), IDH-mutant with 1p/19q co-deletion anaplastic oligodendroglioma (grade III), and pleomorphic anaplastic xanthoastrocytoma (grade III). Because of the therapeutic implications, genetic testing should be performed for IDH, BRAF (epithelioid glioblastoma), MYC (glioblastoma with PNET components), EGFR (small cell and granular glioblastoma) and H3K27M (diffuse midline glioma) [32,34,39]. Histone mutations may vary according to the location of the tumor. K27M mutations in H3F3A (encoding histone H3.3) or HIST1H3B/C (encoding histone H3.1) are very common in tumors arising from the midline and pons, while G34R (or rarely G34V) mutations in H3F3A (encoding histone H3.3) are mostly reported in hemispheric HGGs. In addition, the RTK/RAS/PI3K pathway (e.g., PDGFRA, PIK3CA, PIK3R1, or PTEN) and the p53/Rb pathway (e.g., TP53, CDKN2A, CDK4/6, CCND1-3) are also dysregulated in pediatric HGG [12,13,22,32,36,37,40,41] (Figure 2). 

### 2.4. Ependymoma

Ependymomas are the third most frequently occurring pediatric brain cancers and represent 5–10% of all childhood primary brain tumors [14,17]. Ependymomas are thought to originate from radial glial cells of the ependymal lining of the ventricles and the central canal. More than 90% of ependymomas arise in the infratentorial and supratentorial regions. Histologically, they are classified into four groups: subependymoma, myxopapillary ependymoma, classic ependymoma, and anaplastic ependymoma. Based on histological features, classic ependymoma is further subtyped into three groups: papillary, clear cell, and tanycytic ependymoma. Together, classic and anaplastic ependymoma are the most common subtypes in children [13,14]. Ependymomas can further be subclassified based on location and molecular features predictive of outcome. For example, Group A (PF-EPN-A) and Group B (PF-EPN-B) ependymomas are infratentorial posterior fossa (PF) tumors that differ in their DNA methylation profile. PF-EPN-A tumors are hypermethylated, mostly found in infants and young children, and yield a poorer outcome compared to PF-EPN-B tumors. Supratentorial (ST) ependymomas in children have two major subgroups: RELA fusion-positive (ST-EPN-RELA) ependymoma and YAP1 fusion-positive (ST-EPN-YAP1) ependymoma. ST-EPN-RELA usually harbors the fusion protein of C11orf95 and RELA and, in ST-EPN-YAP1 ependymoma, the transcriptional coactivator YAP1 fuses with other genes such as MAMLD1 and FAM118B, resulting in the upregulation of tumor promoting signaling pathways [12,13,14,15,42,43,44]. Like in other pediatric brain cancers, ependymoma location and molecular characteristics often correlate, highlighting the importance of tumor imaging in early and accurate diagnosis.

## 3. MRI Techniques

### 3.1. Introduction to MRI Modalities

Magnetic resonance imaging (MRI) plays a pivotal role in the initial diagnosis, prognostication, and follow-up of patients with pediatric brain tumors. While conventional MRI is apt in the detection of a brain tumor, it performs poorly in the realm of histopathological and molecular characterization [5]. Functional imaging approaches such as diffusion-weighted imaging (DWI)/diffusion tensor imaging (DTI), perfusion imaging, magnetic resonance spectroscopy (MRS), and magnetic resonance elastography (MRE) offer insights into microstructural and physiological alterations associated with pathological states. These techniques yield enhanced diagnostic performance, superior surgical planning, and improved therapeutic evaluation. 

### 3.2. Diffusion-Weighed Imaging (DWI)

**Figure 3:**
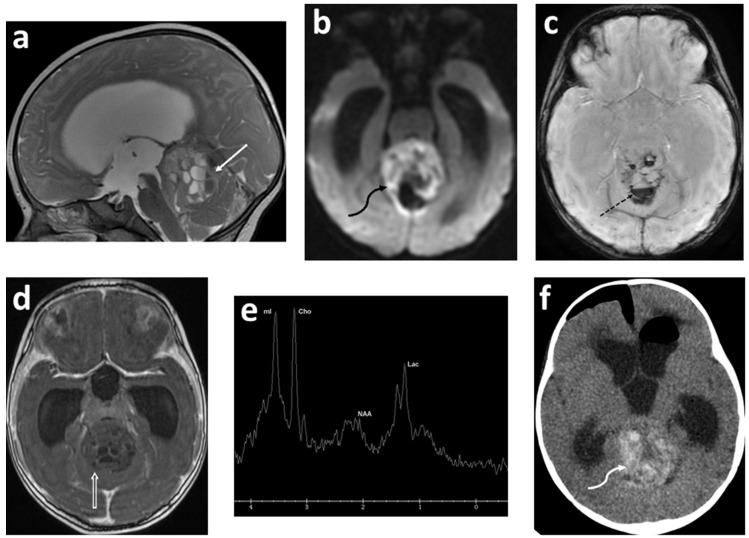
Five-month-old referred for increased head circumference. Sagittal T2 (**a**); axial DWI (**b**); axial SWAN (**c**); axial T1 IR (inversion recovery) post contrast (**d**); short TE 35 millisecond spectroscopy (**e**); and axial non-contrast enhanced CT (**f**) post ventricular shunt placement. There is a large heterogeneous solid ill-defined mass lesion involving the superior and anterior aspect of the vermis (solid white arrow) effacing the fourth ventricle. There are multiple cystic areas within the lesion demonstrating fluid/fluid levels. There is restricted diffusion (curved black arrow) and hemorrhage (dashed arrow). No significant enhancement is identified (open arrow). Spectroscopy demonstrates very high myoinositol (mI), high choline (Cho) and decreased N-acetylaspartate (NAA). There is also elevation of lactate (Lac). CT scan shows high density within the lesion, indicating acute hemorrhage within the lesion (curved white arrow). Pathology confirmed an atypical teratoid rhabdoid tumor (author’s institutional human ethics committee/institutional review board guidelines were followed for anonymized images).

The concept of diffusion-weighted imaging (DWI) stems from the impedance of the free movement of water molecules in the cellular microenvironment which contrasts with the constant random motion of free water molecules (Brownian motion) and is related to their interactions with cellular compartments, including the cell wall and intracellular organelles. The most common method used for DWI is to incorporate two symmetrical motion-probing gradient pulses into a single spin-echo (SE) T2-weighted sequence on either side of the 180° refocusing pulse (Stejskal–Tanner sequence) [45]. If there is no net movement of water molecules, the inherent T2 signal is preserved with a resultant hyperintense signal during DWI. In contrast, the net movement of water molecules along the direction of the applied gradients causes dephasing with T2 signal loss, resulting in a hypointense signal during DWI. A caveat, however, is an increased signal during DWI in areas of the brain with inherently high T2 signal intensity, or the “T2 shine-through”. In such an instance, apparent diffusion coefficient (ADC) maps are helpful to distinguish T2 shine-through from true restricted diffusion. Areas of T2 shine-through will be bright on both DWI and its corresponding ADC map, whereas areas of true restricted diffusion will appear bright on DWI and dark on ADC. One of the most common causes of restricted diffusion is acute ischemic infarction. In the context of tumors, this restriction of water molecules is directly proportional to the cellularity of the tissue [45] (Figure 1, Figure 2 and Figure 3). For instance, in hypercellular tumors such as pediatric CNS embryonal tumors, DWI is helpful in both the diagnosis and detection of recurrence [46]. 

Multiple studies have reported apparent diffusion coefficient (ADC) values to determine cellularity and histologic subtype in pediatric brain tumors [47,48,49,50,51,52,53,54,55]. Hales et al. [5] in their meta-analysis of nine studies (290 patients) demonstrated that pilocytic astrocytoma had significantly higher ADC values compared to both medulloblastoma and atypical teratoid/rhabdoid tumors using both mean and minimum ADC_ROI_ values (Figure 1 and Figure 2). Furthermore, the group suggested a mean ADC_ROI_ threshold of 0.92 × 10^−3^ mm^2^/s for differentiating low-grade from high-grade tumors with a sensitivity and specificity of 100% and 92%, respectively, which was similar to that suggested by Orman et al. Novac et al., in a cohort of 124 patients, suggested a cut off of 0.984 × 10^−3^ mm^2^ s^−1^ for ADC mean to differentiate between ependymomas and medulloblastoma with a sensitivity of 80.8% and a specificity of 80.0% [56]. In clinical practice, increased diffusivity, as suggested by a hyperintense signal on ADC maps, favors a low-grade tumor such as pilocytic astrocytoma, whereas restricted diffusion suggests a high-grade neoplasm such as medulloblastoma. Of note, diffuse midline glioma is an outlier among high-grade tumors with ADC values similar to low-grade tumors. Nevertheless, the development of new diffusion restriction for patients with established diagnosis can help in identifying tumor metastasis, as in the case of embryonal CNS tumors and high-grade gliomas [57,58]. 

Overall, DWI (with ADC data) has a higher utility in differentiating infratentorial tumors as compared to supratentorial tumors [59]. However, challenges and limitations remain, including an overlap of ADC values across various tumor types and grades [60]. Brain tumors can often be accompanied by a varying degree of hemorrhage, which can lead to a confounding effect of lower ADC values and an increase in magnitude of diffusion restriction [61]. Additionally, tumors such as meningioma, DNET, ependymoma and pilocytic astrocytomas can be associated with tumoral calcification, adding a technical challenge to the interpretation of DWI signal and ADC values [62].

The role of DWI and ADC signatures in detecting certain tumor genetic alterations is being investigated in the rapidly evolving fields of radiogenomics and precision medicine [63]. For instance, IDH wild-type gliomas have relatively lower ADC values when compared to IDH-mutant gliomas, and IDH-mutant 1p19q co-deleted subtypes have a significantly lower ADC mean when compared to the 1p19q intact subgroup [64]. In a recent pilot study by Wu et al. of 131 patients with diffuse brain gliomas, a cutoff value of 1.08 for mean relative ADC was able to differentiate IDH wild-type from IDH-mutant groups, with lower ADC averages associated with poorer survival outcomes [65]. The aggressive biology of H3F3A-mutated gliomas that is typically associated with increased cellularity and lower diffusibility has been linked to lower ADC values in children with diffuse intrinsic pontine gliomas [66]. Additionally, children with BRAF V600E-mutant pilocytic astrocytoma were noted to have remarkably lower ADC means than the wild type and relatively unfavorable clinical outcomes [67]. 

### 3.3. Diffusion Tensor Imaging (DTI) and Tractography

The diffusion within white matter is anisotropic, and is hindered to a greater extent in the direction perpendicular to the axons than parallel to them [68]. Diffusion tensor imaging (DTI) can detect such anisotropic diffusion, and needs diffusion to be measured in at least six directions. The orientation of main white matter tracts dictates the direction of the maximum diffusivity of the water molecules. The fractional anisotropy (FA) quantifies such directed diffusion, with values ranging from 0 to 1. An FA value of 0 corresponds to isotropic (undirected) diffusion which graphically translates to a sphere, while an FA value of 1 signifies a totally directed diffusion which would graphically translate to a straight line (and is not evident in biological tissues). Typically, in biological tissues including white matter, the shape of the FA is an ellipsoid. The FA values can be processed into color-coded tractography maps, with blue corresponding to tracts traveling in the superior–inferior plane, red for tracts in the horizontal plane, and green for tracts in the anterior–posterior plane. Fiber tracking can demonstrate neural tracts in 3D. In clinic, DTI has been used to interrogate pathways, such as the corticospinal tract, optic tract, superior longitudinal fasciculus, and arcuate fasciculus [69].

DTI provides in vivo visualization of white matter tracts in the brain and has become an essential tool for pre-surgical planning (Figure 2d–f). DTI can demonstrate local effects of tumors on white matter tracts, such as tract displacement (preserved signal with altered direction/position), edema (decreased but preserved signal with normal orientation), tumor infiltration (decreased signal with tract disruption), and the loss of anisotropic signal (tract disruption). High correlation has been reported between DTI tractography and direct electrical stimulation with a sensitivity and specificity of 92.6% and 93.2%, respectively [69,70,71]. In addition, the acquisition of pre-operative DTI fiber tracking for planning was found to decrease in postoperative deficits from 32.8% to 15.3%, with a longer median survival in patients with high-grade gliomas [72]. Although useful in pre-surgical planning, DTI has multiple limitations such as limited accuracy in areas of crossing fibers, limited reproducibility and accuracy due to a lack of standard analysis protocols, the underestimation of functional white matter tracts in the presence of tumors, and susceptibility to magnetic field inhomogeneity [69].

### 3.4. Functional MRI (fMRI)

Functional MRI is a non-invasive technique to map the eloquent areas of the brain, commonly utilizes task-based paradigms, and is particularly useful in pre-operative surgical planning in patients where tumor and/or resection possibly involves the eloquent areas. Functional MRI exploits the difference in magnetic properties of oxy- and deoxyhemoglobin, with deoxyhemoglobin causing the local dephasing of protons leading to a reduction in signal by creating microscopic field gradients within and in the vicinity of blood vessels [73]. Cerebral blood flow is highly locally regulated in response to oxygen and carbon dioxide concentrations. Performing a task leads to neuronal activation, resulting in increased cerebral blood flow to the corresponding eloquent cortex (neurovascular coupling) which overcompensates for cerebral oxygen utilization. This yields an increase in oxyhemoglobin as compared to deoxyhemoglobin with a resultant decrease in local field inhomogeneity and an increase in MR signal, a phenomenon known as the blood oxygen-level dependent (BOLD) effect [74]. Task-based presurgical BOLD fMRI represents the best established and validated clinical application of fMRI. The typical functional MRI entails performing a task such as finger tapping while the brain is being imaged using a fast T2* sequence such as gradient echo (GRE) echo planar imaging [73]. A typical “block” paradigm has subjects alternate between a resting state and an active task-performing state. The statistical analysis of fMRI data seeks to identify regions of the brain depicting increased or decreased responses in specific experimental conditions. An arbitrary statistical threshold determines which voxel is considered active, and setting this correct threshold is of paramount importance to limit noise and optimize sensitivity [69]. The laterality index, a scoring system, is occasionally used to determine the dominant hemisphere, comparing the total number of active voxels on each side [69,75]. The mapping of motor areas with functional MRI correlates highly with the functional areas identified with direct cortical stimulation with a reported sensitivity and specificity of 95–100% [69,76,77]. However, for language mapping, fMRI sensitivity and specificity range between 37 and 91%, and 64 and 83% [69,76] (Figure 2e,f). 

fMRI has multiple limitations beyond the typical contraindications of MRI, such as pacemakers, claustrophobia, or when the patient is unable to comprehend the request, follow task commands, or pay attention. Neurovascular uncoupling, secondary to abnormal vascularity associated with high-grade gliomas, can interfere with BOLD signals, producing false negative fMRI results and making fMRI unreliable for surgical planning [69,77,78,79]. Similarly, false positive results may also be related to brain plasticity and perilesional edema [69]. In addition, susceptibility artifacts related to blood products and metallic artifacts from surgical hardware may also contribute to suboptimal BOLD signals. 

In children and in patients who are unable to follow commands for task-based fMRI, resting state fMRI (RS-fMRI) offers a viable alternative. RS-fMRI evaluates low-frequency fluctuations in BOLD signals while the subject is at rest and reveals areas of the brain experiencing synchronous activity called resting-state networks (RSNs). Multiple RSNs have been described, including the somatosensory, language, and visual networks. Among the several analysis methods used to identify RSNs, the seed-based method is the simplest and entails the selection of regions of interests (e.g., hand motor area to identify the sensorimotor network), averaging the time course of voxels within the region of interest, and correlating it with the time course of all other regions to determine the connectivity matrix of the brain [80]. Preclinical studies in animal models have shown evidence of tumor-induced changes in resting brain connectivity, as well as intra-tumor connectivity changes in reference to tumor volume and angiogenesis using BOLD RS-fMRI [81]. Additionally, in vivo studies on brain network disruption and alterations in anatomical dysconnectivity (or connectomes) in patients with brain tumors of glial cell origin have contributed to advancing our knowledge of the complex nature of the structural–functional coupling of brain tumors [82,83]. Although still under investigation, multiple studies to date have demonstrated the concordance of RS-fMRI with task-based fMRI or intraoperative mapping, substantiating its clinical utility [69,80,84,85,86,87,88]. Whether the presence of certain connectome patterns can serve as a biomarker of cognitive outcomes of pediatric brain tumors is a subject of ongoing research [89].

### 3.5. Arterial Spin Labeling (ASL) Perfusion Imaging

Arterial spin labeling (ASL) perfusion imaging is a promising alternative to dynamic susceptibility contrast (DSC), and exploits the process of magnetically labelling spins of water protons within the arterial blood by utilizing radiofrequency pulses, which then acts as an endogenous tracer that is delivered to and diffuses throughout the brain parenchyma [90]. Studies have suggested that ASL perfusion imaging can give equivalent useful information, if not superior information, to the DSC-MRI regarding the real-time precision of brain perfusion mapping [91,92]. Moreover, ASL comes with a benefit of avoiding the need for leakage calculation and correction [93]. Of note, ASL allows a more reliable distinction between the progression and pseudo-progression of glial tumors with more than 92% accuracy [94,95]. The non-invasive and non-ionizing acquisition of ASL is specifically beneficial for children and other susceptible patients such as those with decreased renal function. In addition, ASL is particularly well-suited to pediatric brain imaging because children possess higher cerebral water content and generally higher cerebral blood flow, allowing for a higher signal-to-noise ratio and reduced artifacts that might otherwise adversely affect adult studies [90]. The technique of ASL entails three steps: labeling, post labeling delay, and readout. There are three main ASL labeling techniques: continuous arterial spin labeling (CASL), pulsed arterial spin labeling (PASL), and pseudo-continuous arterial spin labeling (pCASL). A detailed review of the principles of ASL is beyond the scope of this paper; interested readers can find more information in another publication by Kerner et al. [90]. Cerebral blood flow (CBF), displayed with color maps, can be quantified in units of mL/100 g/min by placing a region of interest (ROI), or compared as a ratio to a ROI in the contralateral hemisphere or cerebellum.

**Figure 4:**
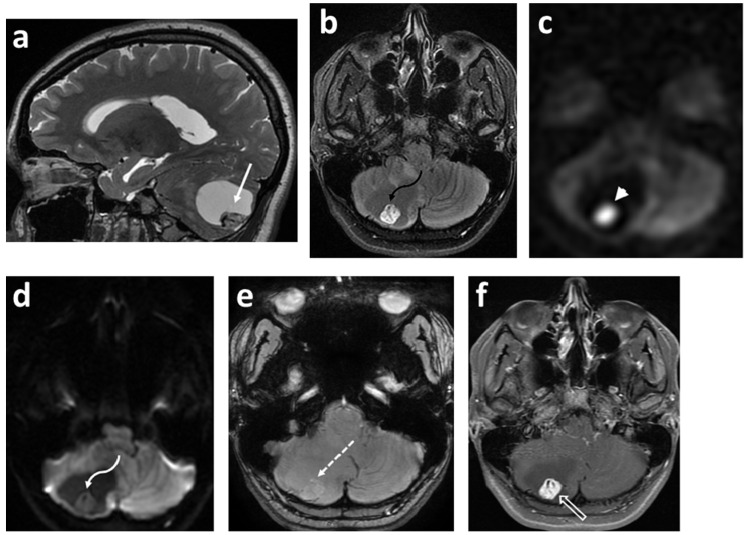
Sixteen-year-old female presented with headaches awakening her from sleep. Sagittal T2 (**a**); axial FLAIR (**b**); axial ASL-PWI (**c**); axial DWI (**d**); axial SWAN (**e**); and axial T1 fat saturated post contrast (**f**) images. There is a large cystic lesion within the right cerebellar hemisphere (solid white arrow) with a solid component within its posterior and inferior aspect (curved black arrow). The solid component of the tumor is hyperintense on T2/FLAIR images with multiple prominent flow voids indicating a highly vascular tumor. There is marked increased perfusion (arrowhead) of the solid component without associated restricted diffusion (curved white arrow). There is no calcification or hemorrhage within the solid component (dashed arrow), with the solid component demonstrating intense enhancement (open arrow) on the post-contrast image. Features are highly specific for hemangioblastoma (pathology proven). Author’s institutional human ethics committee/institutional review board guidelines were followed for anonymized images.

**Figure 5:**
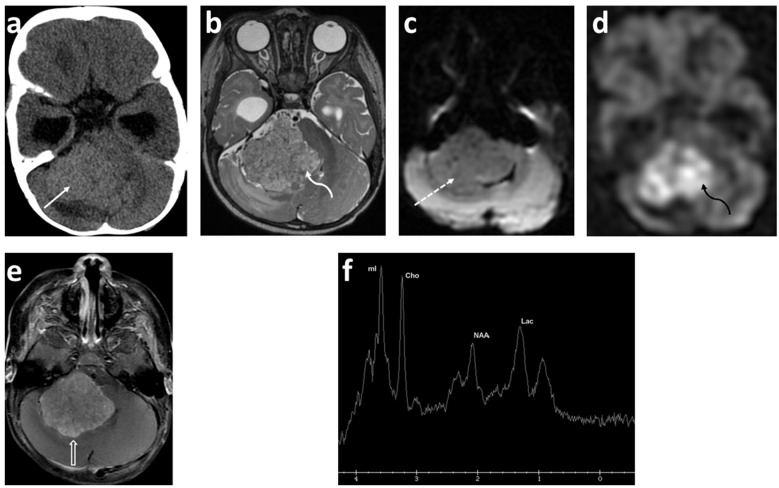
Eight-month-old male referred for bulging fontanelle. Axial CT (**a**); axial T2 (**b**); axial DWI (**c**); axial ASL-PWI (**d**); axial T1 fat saturated post contrast (**e**) images; and short TE 35 millisecond spectroscopy (**f**). CT scan demonstrates mildly hyperdense mass (arrow) with its epicenter in the region of the right foramen of Luschka. The mass is heterogeneously hyperintense on the T2 weighted image (curved arrow). There is no restricted diffusion (dashed arrow). Increased perfusion is identified (curved black arrow) with avid enhancement (open arrow). Spectroscopy demonstrates very high myoinositol (mI), high choline (Cho) and decreased N-acetylaspartate (NAA). There is also elevation of lactate (Lac). Pathology confirmed an atypical choroid plexus papilloma. Author’s institutional human ethics committee/institutional review board guidelines were followed for anonymized images.

Neovascularization is an important pathogenic mechanism in tumor growth and does not correspond with degree of post-contrast enhancement [96]. Perfusion techniques such as ASL are well-suited to evaluate tumoral neovascularization. Hales et al. [5] in their meta-analysis of five studies (252 patients) suggested that although there was considerable overlap, CBF increased with the grade of the tumor [97,98,99,100,101]. ASL can be particularly useful in grading of non-enhancing astrocytomas with nearly 100% sensitivity using the corresponding absolute ratio parameter (TBF max ratio) [102]. Diffuse midline glioma remains an outlier among high-grade tumors, with CBF values similar to low-grade glial tumors. Dangouloff-Ros et al. [97] suggested a cutoff of 50 mL/100 g/min for the differentiation of high-grade from low-grade tumors. This value was most reliable for hemispheric tumors but showed a sensitivity of only 65% for posterior fossa tumors. Yeom et al. found that the maximal relative tumor blood flow of high-grade tumors (grades III and IV) was significantly higher than that of low-grade tumors (grades I and II), with a wider relative tumor blood flow range among high-grade tumors (2.14 ± 1.78) compared with low-grade tumors (0.60 ± 0.29) (*p* < 0.001). Although the relative tumor blood flow did not distinguish individual histology among posterior fossa tumors, relative tumor blood flow was significantly higher for medulloblastoma compared with pilocytic astrocytoma, with a cut-off value of the ratio of tumoral CBF to the brain parenchyma of 0.51, offering a sensitivity of 88% and specificity of 75% [103]. In a cohort of 24 patients, ASL was found to be highly accurate in distinguishing pilomyxoid astrocytoma, a WHO grade II tumor, from pilocytic astrocytoma, a WHO grade I tumor, with 77% sensitivity and 100% specificity using a threshold ratio value of 0.91 [104]. In the cohort of 17 patients reported by Gareton et al, pilocytic astrocytoma with anaplastic features (PAAF) depicted higher CBF values on ASL PWI as compared to classical PA and this finding was applicable to both posterior fossa PA with anaplasia and optic pathways PA with anaplasia. There is a debate on whether the rare phenomenon of the anaplastic variant of pilocytic astrocytoma is applicable only to adults rather than pediatric tumors [105]. In addition, ASL is highly useful in differentiating highly vascular hemangioblastoma from the more common pilocytic astrocytoma with ‘cyst with mural nodule’ morphology [96]. In our experience, the marked increased perfusion within the solid nodule, the ‘light bulb’ sign, is highly suggestive of hemangioblastoma (Figure 4). ASL is promising in distinguishing choroid plexus carcinoma from papilloma, with carcinoma exhibiting significantly higher relative CBF ratio [98,101] (Figure 5). Of note, studies evaluating post-treatment changes in patients with high-grade glioma using ASL are sparse and have small number of subjects, but more evidence is slowly emerging [106]. 

### 3.6. Magnetic Resonance Spectroscopy (MRS)

Magnetic resonance spectroscopy (MRS) offers a non-invasive approach to map the tissue metabolic profile and plays a complimentary role in the pre- and post-therapeutic evaluation of pediatric brain tumors, inherited metabolic disorders, neonatal hypoxic ischemic insults, demyelinating disorders, and infections [107]. ^1^H MR spectroscopy is most widely utilized in clinical practice; however, spectra can be acquired with any nucleus possessing a non-zero spin such as (^15^N) nitrogen, (^13^C) carbon, (^19^F) fluorine, (^23^Na) sodium, and (^31^P) phosphorus [108]. 

In contrast to conventional MRI, ^1^H MRS probes signals of hydrogen attached to various molecules with an output of a plot representing hydrogen nuclei in different chemical environments. The basic “readout” of MR spectroscopy is a spectrum and consists of resonances or peaks representing signal intensities as a function of frequency expressed as parts per million. The *x*-axis of the spectrum is called the ‘chemical shift’ axis and depicts the frequency shift of a proton relative to a universally accepted reference compound (TetraMethylSilane, TMS) at 0 parts per million. The *y*-axis determines the signal intensity; the area under the resonance peak is proportional to the metabolite concentration and to the number of protons contributing to the signal [109]. The typical measurable metabolites on ^1^H MR spectroscopy include N-acetylaspartate (NAA, the neuronal metabolite), choline-containing compounds including glycerophosphocholine and phosphocholine (tCho, marker of cellular membrane turnover), creatine, phosphocreatine, myo-inositol (mIns, the glial metabolite), glutamate and α-aminobutyric acid (neurotransmitters), glutamine, lipids, and lactate [107]. Spectroscopic data in clinical settings are almost exclusively acquired using either localized single voxel or 2D/3D multivoxel (chemical shift imaging) techniques [110]. The vendors commonly provide PRESS (point resolved spectroscopy), STEAM (stimulated echo acquisition mode), and ISIS (image selected in vivo spectroscopy) sequences for data acquisition, and can be acquired at long TEs (TE > 135 ms), and short TEs [110]. The detailed description of data acquisition strategies and post-processing are beyond the scope of this review. However, the prior mentioned sequences differ in the way the radiofrequency pulses and gradient pulses are organized to achieve localization. On a spectrum of the brain from healthy subjects acquired using STEAM and short TE (time to echo), if the four main peaks of the spectrum (mIns, Cho, Cr and NAA) are connected by a manually drawn line, this will form an angle of 45° with respect to the *x*-axis—the Hunter angle. Studies have reported few differences between short TE and long TE regarding the evaluation of brain tumors [111]. For instance, short TE MRS allows a better spatial coverage and a higher resolution in larger and heterogenous tumors with higher lipid content due to necrosis [112]. Over the past few years, the improvement in acquisition strategies such as 2D or, more recently 3D MRS, have resulted in the introduction of and the better understanding of the concept of the onco-metabolite evaluation of D-2 hydroxyglutarate (2-HG) in patients with IDH-mutated gliomas [113,114].

The majority (approximately 60%) of pediatric brain tumors originate within the posterior fossa, with medulloblastoma, pilocytic astrocytoma, diffuse midline glioma, and ependymoma accounting for the overwhelming majority [109,110]. ^1^H MRS plays a complimentary role in the diagnoses of these tumors (Figure 1, Figure 3 and Figure 5). The choline peak in medulloblastoma typically reflects a malignant nature and can distinguish medulloblastoma from its benign mimicker cerebellar dysplastic gangliocytoma, or Lhermitte–Duclos disease [115]. The elevation in creatine and lactate metabolites in children with diffuse fibrillary WHO grade II astrocytoma was found to be helpful in the differentiation from WHO grade I pilocytic astrocytoma [116]. A taurine peak has been consistently identified within the solid components of medulloblastomas, which in conjunction with tumor location and morphology aids in narrowing the diagnosis [109,110,117,118,119,120] (Figure 1). Notable exceptions to this include the desmoplastic/nodular medulloblastoma subtype and tumors with dominant cystic/hemorrhagic components, limiting optimal sampling. In addition, medulloblastomas usually demonstrate relatively higher choline levels corresponding to their high cellularity. Markedly low creatine levels, low myoinositol, diminished choline concentrations reflective of low cellularity, and high lactate are characteristic spectroscopic findings of pilocytic astrocytomas, while ependymomas have relatively high myoinositol levels compared to medulloblastomas and pilocytic astrocytomas [109,110]. 

In the case of supratentorial tumors, a characteristic spectroscopic finding of choroid plexus papilloma is a markedly high ‘sky-rocketing’ myoinositol level, which aids in not only differentiating it from other neoplasms but also from choroid plexus carcinoma [109,110] (Figure 5). With astrocytomas, although accurate grading based on MRS alone is unreliable, evidence suggests that mean choline concentrations increase with grade. Choi et al., using MRS, non-invasively detected ‘oncometabolite’ 2-HG in patients with gliomas and showed its presence concordant with IDH1/2 mutations [121]. This finding was further substantiated by Tietze et al. In their cohort, MRS for 2-HG using a 2 mM threshold accurately identified IDH mutation status in 88.6% of patients with a sensitivity and specificity of 89.5%, and 81.3%, respectively [122]. IDH-mutant diffuse gliomas are exceptionally rare in young children, but are more common in adolescent 14 years of age and above, reaching up to 16% [123]. The presence of 2-HG suggests a better prognosis in adults; it likely portends a worse prognosis in children in comparison to other indolent molecular subtypes (NF1-associated gliomas) [124]. In optic pathway glioma, a characteristic tumor in children with NF1, choline peak and ratio appear to correlate with the tumor grade [125]. Moreover, changes in tumoral NAA/Cho and Lac/NAA can serve as predictors of treatment responsiveness and clinical outcomes in some patients [126]. 

In addition to initial diagnosis, MRS also has a complimentary role in distinguishing post-therapy changes versus tumor recurrence, and neoplastic versus non-neoplastic processes. Overall, pediatric brain tumors tend to have a stereotypical metabolite profile during relapse, making it amenable and plausible to use MRS to assess pattern similarity in comparison with the original tumor, and thus improve the diagnostic certainty of radiological recurrence [127]. Low tCho/Cr or tCho/NAA ratios suggest post-operative changes and radiation necrosis. In contrast, elevated/increasing Cho or tCho/NAA ratios favor residual or recurrent tumors and can serve as prognostic markers [109,110,128,129,130,131]. For instance, a ctCho/tNAA ratio greater than 1.3 was found to have a sensitivity of 100% and a specificity of 94.7% for the detection of glioblastoma relapse [132]. In contrast to neoplastic processes which typically demonstrate elevated tCho and reduce NAA, non-neoplastic pathologies such as abscesses and tuberculous granulomas frequently demonstrate elevated amino acids (isoleucine, leucine, and valine in the 0.95–1.05 ppm range) and lipids [107]. A reduction in myoinositol can be helpful in the differentiation of acute encephalitis from neoplastic processes [110].

### 3.7. Magnetic Resonance Elastography (MRE)

Magnetic resonance elastography (MRE) is a novel, rapidly evolving, phase-contrast-based MRI technique capable of generating quantitative images depicting material properties of tissues such as shear modulus [133], and is inspired by the long-established value of palpation. MRE can be thought of as quantitative, non-invasive palpation. Viscoelastic properties measured with MRE reflect critical information about the microstructural tissue integrity, including the organization and distribution of neurons and axons, glia, and extracellular matrix [134]. The original concept of MR elastography was described by researchers at the Mayo Clinic [135].

**Figure 6:**
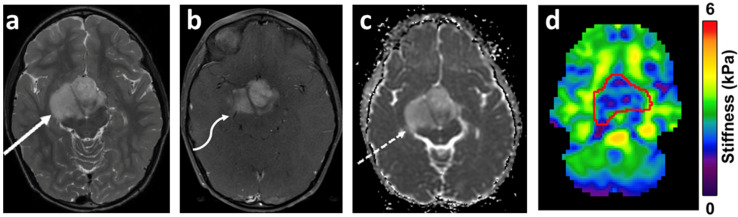
Eight-year-old boy with worsening headaches. Axial T2-weighted image (**a**) demon-strates a hyperintense, heterogeneous mass centered in the optic-chiasmatic, hypothalamic region (arrow). On the post-contrast T1-weighted image (**b**) there is moderate enhancement (arrow), with increased diffusivity on the ADC map (**c**) (arrow). On the stiffness map (**d**), the mass demonstrates heterogenous decreased stiffness (ROI) as compared to the uninvolved white matter. The tumor stiffness measured 2.48 ± 0.70 kPa, while the average brain stiffness excluding the tumor measured 2.83 ± 0.72. Pathology confirmed a ganglioglioma WHO grade I, with Ki-67: 3–4%. Genetic analysis depicted KIAA1549-BRAF fusion. Author’s institutional human ethics committee/institutional review board guidelines were followed for anonymized images.

The technique of MRE entails three basic steps: the generation of shear waves in the brain tissue by an external driver; imaging the propagation of the induced shear waves using a phase-contrast pulse sequence; and the conversion of the displacement data to mechanical properties utilizing inversion algorithms [133]. The shear waves are most commonly generated by an external driver system which has two components: an active driver (vibration source) and a passive driver (acts as an interphase for transmission). The speaker system (active) connects to a pillow-like passive driver via a pneumatic hose. The majority of the brain MRE studies are performed at frequencies ranging from 25 to 62.5 Hz [136], with a frequency of 50 Hz typically used by our group. At 50 Hz, the high amplitude response of the driver is capable of delivering sufficient shear wave motion into the brain parenchyma. MRE obtained at multiple frequencies aids a more complete determination of the rheological behavior of brain tissue [136,137]. Following the generation of shear waves, a phase-contrast-based MR sequence is used to encode the shear wave motion into the MR phase signal using a series of motion-encoding gradients (MEG). The most common pulse sequence used is single-shot echo-planar imaging (EPI) [136], with a typical scan time of 3 min and 15 s. MRE studies are well tolerated with no significant risks (Figure 6). 

To calculate the complex shear modulus, inverse reconstruction algorithms are used to convert the acquired displacement data to mechanical properties. The viscoelastic complex shear modulus (G*) has two components expressed as the real and imaginary components of the complex quantity: G* = G′ + iG″. G′ is the shear stiffness also referred to as the elasticity or storage modulus and is mainly reflected in the wavelength of the propagating shear waves. An increase in stiffness results in shear waves travelling faster with a resultant increase in wavelength. G″ is the shear viscosity or loss modulus and is reflected in the attenuation of the waves as they travel through a medium. Commonly reported stiffness (μ) and damping ratio (ξ) are calculated from the viscoelastic complex shear modulus. The detailed review of the principles underlying MRE is beyond the scope of this paper. 

Pre-operative assessment of brain tumor stiffness has potential implications in surgical planning in regard to the determination of surgical approach, instrument selection, and resection strategy, and has been linked to postoperative complication risk and the extent of resection [138,139]. There is a significant dearth of studies assessing the role of MRE in pediatric brain tumors. However, multiple studies have evaluated the role of MRE in various brain tumors in adults including gliomas, meningioma, pituitary adenomas, and metastases [140,141,142,143,144,145]. Pepin et al. studied the relationship of tumor stiffness (G*) with tumor grade and isocitrate dehydrogenase 1 (IDH1) mutation status and reported an inverse relationship between glioma grade and stiffness. Interestingly, within their cohort, the reported IDH1 wild-type tumors (n = 12) depicted lower stiffness as compared to IDH1 mutant tumors [142]. Murphy et al., in their cohort of 12 cases of meningiomas, concluded that the ratio of tumor stiffness to the stiffness of surrounding brain tissue correlated positively with the surgeon’s qualitative assessment of the tumor, utilizing a 5-point scale ranging from 1 (soft: 100% removable by suction) to 5 (hard: uniformly hard, the majority requiring ultrasonic aspiration) [145]. Whether the inclusion of MRE data in ever-evolving radiogenomics models could improve the accuracy of phenotypic–genotypic correlation imaging for brain tumors is currently unknown, though it seems that it could add potentially important prognostic information and improve clinical decision-making.

### 3.8. Amide Proton Transfer (APT)-Weighted Imaging

Amide proton transfer (APT)-weighted imaging is a type of chemical exchange saturation transfer (CEST)-based imaging with a heightened sensitivity to endogenous mobile proteins and peptides, and it has shown promise in characterizing brain tumors [146,147,148,149]. The difference (increase) in the contents of mobile protons and peptides in the tumor tissue relative to the surrounding parenchyma leads to an increased APT signal [150], with high-grade/malignant brain tumors exhibiting a high degree of protein content. There is an expanding role for APT-weighted imaging in the detection, grading, evaluation of recurrence versus post-treatment effects, and the identification of the genetic markers of brain tumors. Jiang et al. reported the APT signal as a valuable biomarker aiding the pre-surgical differentiation of primary central nervous system lymphomas (PCNLs) from high-grade gliomas [151]. Similarly, Yu et al. reported the high accuracy of APT-weighted imaging (82.5%) for distinguishing solitary brain metastasis (SBM) from glioblastoma (GBM) [148]. Interestingly, although the APTw values in the tumor core were not significantly different between the SBM and GBM groups, the APTw values in the peritumoral brain zone (PBZ) were significantly lower in the SBM group than in the GBM group. Suh et al., in a recent meta-analysis, reported the pooled sensitivity and specificity of APT-weighted imaging for differentiating low-grade and high-grade tumors to be 88% and 91%, respectively [152]. 

### 3.9. Radiomics and Radiogenomics

Radiomics is a growing field that involves converting radiological images into high-dimensional mineable data which can be further used to create machine learning models predict clinical outcomes. In contrast to visual assessment—which offers information on macroscopic tumor characteristics such as size, location, enhancement, and diffusion restriction—computer vision has potential to uncover clinically significant high-dimensional features concealed to the human eye. The field of radiogenomics may potentially aid in the non-invasive histological and genetic classification of brain tumors. 

Despite significant advances in imaging approaches, challenges remain for the accurate non-invasive prediction of tumor grade/subtype, post-treatment response evaluation, and prognostication. Opportunities for improvement are numerous. Furthermore, advances in the understanding of the molecular pathogenesis of gliomas have not been optimally translated into the recognition of characteristic imaging phenotypes. Stereotactic brain biopsy remains the reference standard for histological and genetic classification despite being invasive, underpinning the need for the identification of imaging surrogates. In addition, there is a greater need for incorporating data from various advanced functional imaging techniques such as DWI/DTI, MRS, MRE, PWI and radiotracer imaging into diagnostic pipelines. Multiple recent studies have shown the prognostic value of multiparametric magnetic resonance imaging in the context of brain tumors [153,154,155,156,157,158,159]. 

Radiomics workflow entails multiple steps such as image acquisition and reconstruction, pre-processing, the identification of regions of interest, feature extraction, quantification, selection, and building predictive/prognostic models with machine learning [153]. The features extracted in radiomics are morphological (perimeter, elongation factor, and surface curvature attributes), textural (Gabor descriptors, histograms of oriented gradients (HOG), and local binary patterns (LOG)), and functional. Functional radiomics features are a new class of markers aimed at capturing physiologic properties such as angiogenesis (properties of feeding vessels such as convolutedness and density) [153]. Radiogenomics is an advancing field of radiomics which involves associating genetic mutations and pathways with distinct imaging phenotypes [160].

Multiple studies have reported the application of radiomics/radiogenomics pipelines in both adult and pediatric neuro-oncology. Tian et al., using the texture analysis of an multiparametric MRI, reported an accuracy of 96.8% for classifying LGGs from HGGs and 98.1% for classifying grade III from grade IV [161]. In pediatric low-grade gliomas, patients with BRAF fusion and neurofibromatosis type 1 have favorable outcomes compared to those with BRAF V600E mutation, particularly in association with cyclin-dependent kinase inhibitor 2A (CDNK2A) deletion. Wagner et al., using radiomics features such as histogram, shape, and texture to predict BRAF status, reported an area under the curve (AUC) of 0.75 on the internal validation cohort, and an AUC of 0.85 for the external validation cohort [162]. A recent multi-institutional study in children with diffuse intrinsic pontine glioma demonstrated that a combined model incorporating clinical variables and radiomics features was superior in predicting overall survival as compared to clinical variables alone, with a concordance index (CI) of 0.7 and 0.59 in the training and testing datasets [163].

### 3.10. Response Evaluation of Pediatric Brain Tumors

Magnetic resonance imaging is heavily relied upon for determining responses to therapy. Over the years, the criteria and endpoints used to assess response to therapy have evolved. Until 2010, most neuro-oncologists and neuro-radiologists relied upon MacDonald and the Response Evaluation Criteria in Solid Tumors (RECIST). MacDonald criteria were preferentially used because it was thought that the use of two orthogonal diameters (2D) might be advantageous in comparison to the single longer diameter (1D) used in the RECIST criteria. Both criteria had significant limitations, as they addressed only the contrast-enhancing portion of the tumor [164]. The Response Assessment in Neuro-Oncology Working Group (RANO) is an international, multidisciplinary effort working to standardize the assessment of tumor response radiographically in conjunction with clinical evaluation. This group addressed the limitations of earlier response assessment criteria and most notably concluded that contrast enhancement is non-specific and does not reflect therapeutic response [165]. The RANO working group is subdivided into specific groups: RANO-HGG, RANO-LGG, RANO seizures, RANO-BM, RANO-LM, iRANO, NANO, RAPNO, SPINO, RANO meningioma, RANO PET, the RANO surgery group, RANO steroid, and RANO patient-reported outcomes. Given that pediatric brain tumors are molecularly and clinically different than adult brain tumors, the Response Assessment in Pediatric Neuro-Oncology (RAPNO) has outlined criteria for response assessment in pediatric high-grade and low-grade gliomas [166,167,168]. The RAPNO committee’s goal is to establish standardized protocols that can be applied internationally without substantial confounding factors. These consensus recommendations, however, need to be validated in prospective clinical trials.

## 4. Positron Emission Tomography (PET) Imaging

### 4.1. Introduction to PET Imaging

Positron emission tomography (PET) is a powerful imaging modality aiding the real-time quantification of various biological processes such as enzyme and receptor levels, blood flow, and metabolic rates, utilizing a radiolabeled molecule (radiotracer). PET uses radioactive isotope-labeled compounds to aid in diagnosis, monitoring disease progression, and identifying response to treatment. Due to the small carrier mass of the labeled molecules, the PET imaging agents are also called radioactive tracers or radiotracers. The radiotracers emit positively charged anti-electrons called positrons. The positron travels a short distance and annihilates with an electron to give two photons. The photons have an energy of 511 kiloelectron volts (keV) and travel at almost 180^o^ to each other. Multiple detectors surrounding the subjects within the PET scanner detect these high-energy photons. The images are then reconstructed using computer software to localize the origin of the photons in the body by a back-projection method. The PET radiotracer injected during imaging procedures undergoes whole-body distribution, accumulation, and excretion. The positron decay of the radiolabeled molecule aids in the detection of the tracer location and its magnitude of accumulation.

PET imaging has a very high sensitivity and specificity. Isotopes as low as 100 picomolar levels can be detected in the target tissues; this microdose property often leads to a negligible physical effect on the living subjects. Thus, PET is ideal for studying the mechanism of action and whole-body distribution independent of any physiological consequences [169]. In addition, PET scans possess millimeter-level spatial resolution, which can be used in preclinical research and clinical investigation for drug development and daily clinical practice. So far, PET is the most sensitive and specific technique for probing functional changes at a molecular level [170]. 

### 4.2. Investigational Probes

The use of PET is rapidly growing in the care of children and pediatric-related research. The development of fused imaging modalities, such as PET/computed tomography (CT) and PET/magnetic resonance imaging (MRI), has further advanced pediatric research and clinical applications. FDG is the predominant PET radiotracer for pediatric brain tumors. FDG continues to provide critical information aiding in diagnosis, staging, grading, prognosis, evaluating recurrence, treatment planning, and assessing the treatment response of pediatric brain tumors [171,172]. However, FDG PET is limited by high uptake in the cerebral gray matter of the normal brain, leading to a low ratio of tumor to normal tissue uptake [173]. There is also nonspecific uptake associated with inflammatory CNS lesions and, therefore, false positives have been noted in neuroinflammatory conditions [173]. Furthermore, most low-grade brain tumors display low metabolism for FDG, and tumors cannot be readily detected with this tracer [174]. Therefore, there is an increasing interest in non-FDG-PET tracers to provide complementary information for pediatric brain tumors.

In addition to commercially available FDG, amino acid-based radiotracers are the most often used PET imaging agents in the evaluation of pediatric brain tumors. Amino acid PET has significant advantages over FDG PET due to its high uptake in brain tumors, including low-grade gliomas with superior tumor-to-nontumor ratios, and low uptake in normal brain, resulting in more specificity for detecting brain tumors and the more precise delineation of tumor boundaries [175]. In a retrospective study of 17 children aged 2–16 years with brain tumors, Driever et al. reported that MRI identified new lesions during or at the end of the cancer treatment, demanding further management. The brain tumors included ependymoma, medulloblastoma, low-grade glioma, high-grade glioma, germ cell tumor, and choroid plexus tumor. O-(2-[^18^F]-fluoroethyl)-tyrosine ([^18^F]FET) imaging was performed for compassionate use to help with clinical decisions. PET scans were performed 10 min after the intravenous injection of 70–200 MBq [^18^F]FET in a weight-adapted fashion. The results showed that [^18^F]FET readily differentiated tumor tissues from post-therapeutic changes in 16 of 17 patients. Among them, lesions in 13 patients were tumor tissues, and 3 were post-therapeutic changes. While the study had a limited sample size, it demonstrated the benefit of using [^18^F]FET PET as a promising diagnostic tool in pretreated children and adolescents with equivocal brain lesions [176]. Dunkl et al. used [^18^F]FET on 26 children and adolescents (1–18 years old) with newly diagnosed brain tumors. The results showed that when the maximum tumor-to-background ratio reached 1.7 or above, [^18^F]FET detected tumor tissues with the highest accuracy (77%). In addition, the study found that [^18^F]FET added valuable information for diagnosing tumor progression or recurrence when evaluating response to chemotherapy [177]. Misch et al. reported that [^18^F]FET correctly diagnosed 20 out of 24 evaluable patients with various brain tumors; only two pretreated patients received false positive results [178]. 

^18^F-3,4-Dihydroxyphenylalanine ([^18^F]DOPA) was approved by the U.S. Food and Drug Administration (FDA) for the early detection of suspected parkinsonian syndromes (PS) in adult patients in 2019. [^18^F]DOPA has been widely used for imaging pediatric brain tumors. Morana et al. used [^18^F]DOPA combined with conventional MRI to assess 13 patients with infiltrative astrocytomas (8 boys and 5 girls). The results showed that [^18^F]DOPA uptake displayed a heterogeneous distribution in all positive cases. Significantly higher tumor uptake in high-grade tumors compared with low-grade lesions was observed. Furthermore, [^18^F]DOPA uptake correlated well with progression-free survival [179]. The same team used [^18^F]DOPA combined with MRS to assess supratentorial infiltrative gliomas in 27 pediatric patients (4–17 years old) and found significant positive correlation between the MRS data and [^18^F]DOPA parameters, with the strongest correlation between [^18^F]DOPA uptake and the Cho/NAA ratio. [^18^F]DOPA uptake and proton MRS ratios were significantly higher in high-grade tumors than their low-grade counterparts. [^18^F]DOPA uptake correlated with progression-free survival and overall survival [180]. Later on, Morana et al. used [^18^F]DOPA PET/CT and fused PET/MRI to evaluate striatal involvement in children with primary, residual, or recurrent gliomas. In the 28 pediatric patients (1–18 years old), [^18^F]DOPA PET/MRI showed 100% accuracy, 93% sensitivity, 89% specificity, and 100% positive predictive value. [^18^F]DOPA PET/MRI also showed significantly higher accuracy than [^18^F]DOPA PET/CT. Basal ganglia involvement with physiological striatal ^18^F-DOPA uptake did not seem to severely interfere with imaging interpretation. The use of fused ^18^F-DOPA PET/MRI further improved the accuracy compared to the single imaging modality [181]. Gauvain and colleagues evaluated the response to bevacizumab antibody therapy with ^18^F-DOPA in six children with relapsed gliomas (8–12 years old). The results showed ^18^F-DOPA PET/MRI was well tolerated by all patients and visualized all tumors. The percent of metabolic tumor volume at the 4-week scan correlated with progression-free survival [182]. More recently, Morana et al. also used [^18^F]DOPA combined with DWI and ASL MRI sequences to assess diffuse astrocytic tumors in 26 pediatric patients (2–17 years old). The study found a significant correlation between maximal CBF, DWI-derived minimum apparent diffusion coefficient, and [^18^F]DOPA uptake. The combined PET and MRI increased prognostic ability. There was also a significant difference in [^18^F]DOPA uptake between low- and high-grade tumors [183]. 

Rosenfeld et al. used both FDG and L-(methyl-[^11^C])methionine ([^11^C]MET) to evaluate diffuse intrinsic brainstem gliomas in 30 children (2–13 years old). Both tracers showed focal and generalized metabolic activity, but no correlation with survival was observed. Patients with negative scans for both tracers had a longer survival rate than those with positive scans. However, patients with negative FDG scans and positive [^11^C]MET scans had the shortest survival time [184]. Laser reported [^11^C]MET PET in 10 pediatric patients (5–19 years old) with craniopharyngioma prior to proton therapy. The maximum standardized uptake value (SUV_max_) showed that [^11^C]MET had a high tumor to background ratio compared to FDG [185]. In another study involving 65 patients (12 children, 53 adults, 2–83 years old), Laukamp et al. found that [^11^C]MET PET was useful in delineating and grading the gliomas. The authors used binary logistic regression models to differentiate WHO tumor types and grades with an accuracy of 80%. The results showed that [^11^C]MET PET could visualize tumor activity where MRI findings were negative [186]. Phi et al. used [^11^C]MET in the differential diagnosis of pediatric epilepsy in patients with focal cortical dysplasia (FCD), dysembryoplastic neuroepithelial tumor (DNET), or ganglioglioma. Among the thirty patients studied (11 months to 17 years old), FDG was non-contributary to the differential diagnosis, and there was no significant difference in the lesion-to-gray matter ratio between the groups; however, [^11^C]MET PET identified a significant difference among the diseases, and correlated well with the pathological spectrum in pediatric patients with lesional epilepsy [187]. Rheims et al. summarized [^11^C]MET scan results in 77 patients with focal epilepsy related to a chronic progressing brain tumor found on MRI. Fifty-two of the patients (3–39 years old) had definite histopathology. Semiquantitative analyses showed that a normal [^11^C]MET uptake was correlated with DNET, while moderate and high tumor uptake indicated gliomas or gangliogliomas. [^11^C]MET could distinguish DNETs from other tumor types with 90% specificity and 89% sensitivity [188]. Pirotte et al. summarized 400 consecutive pediatric brain tumors treated between 1995 and 2005. Among them, 126 children underwent either FDG or [^11^C]MET scans in the diagnostic workshop. The team found that PET imaging was beneficial in 30% of all pediatric brain tumor patients, particularly when there was a debate between surgery and conservative MR follow-up or poor MRI quality for the choice between biopsy and resection [189]. 

α-[^11^C]-methyl-L-tryptophan ([^11^C]AMT) has been investigated for mapping serotonin synthesis, detecting epileptogenic lesions, and guiding epilepsy surgery [190,191,192,193,194]. [^11^C]AMT also finds extensive application in pediatric brain tumors. Peng et al. reported the utility of [^11^C]AMT for assessing an isolated optic pathway glioma (OPG) in a 16-year girl. Twenty minutes after the administration of [^11^C]AMT, PET images were reconstructed with co-registered MRI images. The results showed [^11^C]AMT had increased uptake by OPG, while MRI was inconclusive for morphological changes. After chemotherapy, the tracer uptake was dramatically decreased. [^11^C]AMT PET subsequently identified a new tumor lesion when the patient developed a vision problem, with no interval morphological changes. Upon the completion of external beam radiotherapy, the vision was significantly improved. [^11^C]AMT may be helpful for monitoring the progression and response to treatment for OPGs [195]. Luat et al. reported an 11-year-boy with a large oligodendroglioma of the right temporal lobe. The boy presented with attention-deficit hyperactivity disorder (ADHD), obsessive compulsive disorder (OCD), and stimulant-induced tic disorder, and later on seizures. The [^11^C]AMT PET scan showed asymmetric uptake in the basal ganglia and high uptake in the tumor. After surgery, the child was ADHD-free and with only minimal symptoms of OCD [196]. The same group evaluated indoleamine 2,3-dioxygenase (IDO) expression (a rate-determining enzyme in the tryptophan metabolism) in resected brain tumor specimens from 15 patients (4–67 years old). The patients underwent [^11^C]AMT PET scans before tumor resection. The results showed that six of seven low-grade tumors presented IDO immunoreactivity; this was only observed in one of eight high-grade tumors. [^11^C]AMT metabolic rates correlated well with IDO expression levels. Therefore, [^11^C]AMT PET helps to identify brain tumors with differential IDO express levels [197]. Juhasz et al. reported the utility of [^11^C]AMT PET to measure the primary brain tumor uptake of tryptophan and standardized uptake values (SUVs) in 40 patients (1.5–67 years old, 16 children). The results showed that 95% of grade II to IV gliomas and glioneuronal tumors, including recurrent and residual tumors, presented with increased SUV, while 77% showed decreased or normal FDG uptake [198]. In a subsequent study, the team applied [^11^C]AMT to study temporal lobe DNETs in 11 children (2–18 years old). The results showed that [^11^C]AMT was particularly sensitive for detecting DNETs and had much higher tumor-to-cortex ratios than FDG. The accumulation of [^11^C]AMT in DNETs was driven by the L-type amino acid transporter 1. The study also found that [^11^C]AMT uptake extended to the extratumoral cortex, indicating [^11^C]AMT may detect the extratumoral epileptogenic cortex and predict the likelihood of recurrent seizures [199]. Juhasz et al. examined [^11^C]AMT PET to detect low-grade gliomas and glioneuronal tumors in 23 patients (3–57 years old). The team used tumor dynamic PET, blood input data, and metabolic rates to measure the unidirectional uptake rate and volume of distribution (VD). Increased tumor/cortex ratios or VD ratios were observed with [^11^C]AMT PET. [^11^C]AMT PET could identify most low-grade gliomas and DNETs with high tracer uptake, even when there was no enhancement on MRI for these tumors [200]. Bosnyák and colleagues studied tryptophan metabolism in meningiomas and compared it with gliomas using [^11^C]AMT for 47 patients (10–91 years old). Kinetic analysis showed that [^11^C]AMT could differentiate grade I meningiomas from their grade II/III counterparts with a k3’ tumor-to-cortex ratio. Furthermore, [^11^C]AMT could also differentiate meningiomas from low-grade and high-grade gliomas [201].

In addition to amino acid tracers, choline-based radiotracers have also been used in the PET imaging of pediatric tumors; these tracers were initially developed to image prostate cancers [202]. Fraioli et al. studied astrocytic brain tumors in 12 pediatric patients with ^18^F-fluoroethylcholine (^18^F-choline) and MRI. The results showed a negative correlation trend between SUV_max_ and the mean ADC value; a positive correlation between SUV_max_ and tumor size was observed. A concordant decrease in the mean size of the tumor, SUV, and an increase in ADC suggested a correlation between cellularity and metabolic activity [203]. Later, the same group used ^18^F-choline to detect residual tumor cells in patients with intracranial non-germinomatous germ cell tumors. Tumor viability correlated well with ^18^F-choline uptake. Viable tumors had persistent tracer uptake, while there was no tracer uptake in patients with negative histology post-treatment [204].

Other non-small molecular radiotracers have been investigated for imaging pediatric brain tumors. In a case report, Arunraj et al. found that the ^68^Ga-labeled peptide [^68^Ga]DOTANOC could detect medulloblastoma recurrence in an adolescent patient. The neuroendocrine tumors, including paraganglioma, medulloblastoma, and meningioma, had a high expression of somatostatin receptors, and the peptide possessed a good binding affinity targeting the receptor. In addition, the radionuclide ^68^Ga has a half-life of 68 min and is readily available through the [^68^Ge]/[^68^Ga] generator. The radiolabeling can be applied using the bifunctional chelator (BFC) to bridge ^68^Ga and the biomolecules [205]. In another case report, Veldhuijzen van Zanten, et el. used a ^89^Zr-labeled antibody ^89^Zr-bevacizumab for PET imaging for a 12-year-old girl with DIPG. The results showed heterogeneous uptake between tumor lesions with ^89^Zr-bevacizumab imaging. In addition, the tracer uptake correlated with the tumor histological analysis at postmortem examination [206].

Overall, most investigational PET tracers for pediatric brain tumors are amino acid-based derivatives. With these radiotracers, the PET imaging of pediatric brain tumors has several advantages: low background uptake, high tumor target-to-nontarget ratios, optimized biopsy targeting, and enhanced accuracy in detecting tumor recurrence. Furthermore, the natural amino acid analog with minimal carrier mass is expected to possess negligible toxicity for the radiolabeled radiotracers [207]. Peptide and antibody PET tracers for pediatric brain tumors are emerging with promising results, while more studies with a large sample size are needed to validate the findings. The integration of PET/MRI decreases radiation dose, improves motion correction, and facilitates a combined exam, adding the most significant value for PET utilization in pediatric brain tumors. 

## 5. Single-Photon Emission Computed Tomography (SPECT) Investigative Tracers

SPECT, a nuclear medicine technique, has been in common use since the 1990s. Compared to PET, SPECT is much more available, cheaper, and widely used. However, SPECT typically needs a longer scan time, has a lower resolution, and is prone to artifacts and attenuation [208,209,210]. It has been mainly used to estimate perfusion and cerebral blood flow, and studies in pediatric brain tumors have been relatively scarce. Most of the studies were reported in the late 1980s and 1990s before the widespread availability of advanced MRI and PET imaging. Thallium-201 (^201^TI, half-life 73 h) and ^99m^technetium (^99m^Tc, half-life 6 h) are the two common gamma-emitting isotopes used for pediatric brain tumors. ^201^TI is highly specific for studying the metabolic activity of pediatric brain tumors. In a study involving 19 children with brain tumors, the authors compared ^99m^Tc-2-methoxy-isobutyl-isonitrile (^99m^Tc-MIBI) with ^201^TI-based agents. The results showed that ^99m^Tc-MIBI had a better signal-to-noise ratio with a sensitivity of 67% and a specificity of 100% [211]. In another prospective and comparative study of 24 pediatric brain tumors with ^201^TI SPECT and MRI, ^201^TI uptake was shown in 14 lesions. The study found that ^201^TI imaging underestimated tumor burden. Furthermore, the accumulation of ^201^TI did not correlate with tumor type, grade, or malignancy. The authors concluded that ^201^TI SPECT did not provide helpful information over gadolinium-enhanced MRI [212]. Kirton et al. reported using ^99m^Tc-MIBI SPECT to evaluate 20 children with brain tumors. The results showed that specific tumors, including brain stem glioma, glioblastoma, pilocytic astrocytoma, and choroid plexus carcinoma, had ^99m^Tc-MIBI uptake. However, no tracer uptake was observed in DNET or medulloblastoma. The study concluded that ^99m^Tc-MIBI might be used for the differential diagnosis of various tumor types [213]. In three pediatric cases of temporal lobe seizure due to calcified glioma, ^201^TI chloride and ^123^I-isopropyl iodoamphetamine (^123^I-IMP) showed high uptake in one tumor. Both tracers provided further information on tumors, including malignancy. High ^201^TI chloride and ^123^I-IMP uptake correlated with high-grade gliomas [214]. Amino acids labeled with SPECT isotopes have also been reported to image pediatric brain tumors. For example, L-3-[^123^I]iodo-α-methyl tyrosine (IMT)-SPECT has been used to image pediatric pilocytic astrocytoma. IMT-SPECT displayed high tumor uptake in 7 out of 16 cases [215], and was comparable with FDG in 7 out of 10 patients. The results demonstrated that glucose metabolism and amino acid transporters were increased in the tumors. Overall, SPECT investigative tracers for pediatric brain tumors are relatively rare due to their lower resolution and longer scan time. However, they are complementary to PET and MRI imaging modalities, and are becoming a standard of care for pediatric imaging [216].

## 6. Multimodality Fusion Techniques and Treatment Planning

Computed tomography (CT) simulation has been the cornerstone of radiotherapy planning for the past several decades and is utilized to determine target volumes, including gross tumor volume (GTV), clinical tumor volume (CTV), and planning target volume (PTV), which are contoured onto CT images. The critical organs at risk such as the optic nerve, optic chiasm, hippocampi, brain stem, spinal cord, and cochleae are also contoured to minimize toxicity. Additionally, CT simulation provides a spatial electron density map (calculated from CT-derived Hounsfield units) which is vital for radiation dose calculation [217,218]. However, CT imaging is critically met with poor soft tissue contrast within the neuroaxis. One of the most common strategies to circumvent this limitation is to fuse or co-register MRI sequences providing superior soft tissue contrast and resolution with CT simulation imaging. The most commonly utilized MRI sequences to determine tumor volumes include post contrast T1-, and T2/FLAIR-weighted sequences.

Multiple studies have demonstrated that the utilization of MRI co-registration results in significant changes in contoured target volumes and at-risk organs, with an increasing number of institutions pursuing the use of dedicated MRI simulators [217,219,220]. In children, MRI-only RT planning alleviates the need for multiple episodes of sedation, consolidating simulation to a single scan with improved workflow. However, MRI alone is not sufficient for RT planning due to absence of electron density information for radiation dose calculation. A viable solution to derive electron density information from MRI data includes the generation of synthetic CT images. Multiple approaches have been described to generate synthetic CT images, such as atlas-based deformable image registration, MR bone imaging-based tissue classification or segmentation, and machine learning-based convolutional neural networks (CNNs) [218].

There is increasing interest in incorporating advanced/functional MRI techniques into treatment planning and response evaluation, such as DWI and MRS [217,220]. MRS, given its ability to provide metabolic data, including those related to cell turnover and hypoxia, can potentially impact treatment planning and response [217]. The incorporation of functional imaging techniques into radiotherapy planning and follow-up offers the potential for improving clinical outcomes.

## 7. Conclusions

Brain and central nervous system tumors are the most lethal and the most common solid tumors in children. The type, location, and molecular signature of brain tumors in children differ from adults. Furthermore, tumor incidence, type and location vary in children by age and other factors. Children present with a variety of symptoms, making early identification and accurate diagnosis especially challenging. Neuroimaging plays a vital role in diagnosis, treatment planning, and the monitoring of disease progression and response to therapy. Standard MRI provides information for tumor detection. Advanced MR imaging techniques, including diffusion-weighed imaging (DWI), diffusion tensor imaging (DTI), functional MRI, arterial spin labeling (ASL) perfusion imaging, MR spectroscopy (MRS), and magnetic resonance elastography (MRE), provide additional and improved structural and functional information, and when combined with positron emission tomography (PET) or single-photon emission computed tomography (SPECT), provide functional information on tumor metabolism and physiology through the use of radiotracer probes. Radiomics and radiogenomics offer promising insight into the prediction of tumor subtype, post-treatment response to treatment, and prognostication. Advanced neuroimaging in conjunction with evolving criteria for the evaluation of response to treatment, as well as the emergence of high-quality portable imaging devices [221,222,223], will aid in improved care and treatment for children with the currently most lethal form of pediatric cancer: brain tumors.

## Figures and Tables

**Table 1:** Pediatric brain tumors—an overview.

Family	Tumor Type	Additional Subtyping Based on Molecular Alterations	Frequent Molecular Alterations (*)
**CNS embryonal tumors**			
Medulloblastomas	WNT-activated		*CTNNB1;* often in conjunction with monosomy chromosome 6, *DDX3X, SMARCA4*, and *TP53* mutations
	SHH-activated (wildtype *TP53*)	SHH-1, SHH-2, SHH-4	*PTCH1, SUFU, SMO*
	SHH-activated with mutant *TP53*	SHH-3	*TP53, MYCN* amplification*, GLI2* amplification
	Non-WNT/non-SHH (Group 3 and Group 4)	Subtypes 1-8	*MYC* or *MYC/MYCN* amplification, *GFI/GFI1B* alterations, *OTX2*, *CDK6* or *SNCAP1* amplifications; isochromosome 17; *PRDM6*, *KBDBD4* mutations
Atypical teratoid/ rhabdoid tumors	ATRT-TYR, ATRT-SHH, ATRT-MYC		*SMARCB1*, *SHH*, *NOTCH*, loss of 22q, tyrosinase overexpression, *MYC* activation, *HOXC*
**Gliomas, glioneuronal and neuronal tumors**			
Diffuse high-grade gliomas	Diffuse midline glioma, H3 K27-altered	H3.3 K27-mutant; H3.1 or H3.2 K27-mutant; H3 wildtype with *EZHIP* overexpression; *EGFR* (and H3 K27) mutant	Histone 3 mutations, *TP53*, *PPM1D*, *PDGFRA*, *PIK3CA*, *PIK3R1*, *PTEN* mutations, *EZHIP* overexpression, *EGFR* mutations
	Diffuse hemispheric glioma, H3 G34-mutant		Histone 3 mutation, *TP53*, *ATRX* mutations
	Diffuse pediatric-type high-grade glioma, H3-wildtype and IDH-wildtype	RTK1; RTK2; *MYCN*	Enriched for *PDGFRA*, *EGFR* or *MYCN* amplification
	Infant-type hemispheric glioma	*NTRK*-altered; *ROS1*-altered; *ALK*-altered; *MET*-altered	*NTRK1/2/3* fusion; *ROS1* fusion; *ALK* fusion; *MET* amplification/fusion
Diffuse low-grade gliomas	Diffuse astrocytoma	*MYB*-altered; *MYBL1*-altered	*MYB* fusion or *MYBL* fusion commonly with *PCDHGA1*, *MMP16* and *MAML2*
	Angiocentric glioma		*MYB* alterations, commonly fused with *QKI*
	Polymorphous low-grade neuroepithelial tumor		MAPK pathway–*BRAF* pV600E, fusions with *FGFR2* or *FGFR3*
	Diffuse low-grade glioma, MAPK pathway-altered	*FGFR1* tyrosine kinase domain-duplicated; *FGFR1* mutant; *BRAF* pV600E-mutant	MAPK pathway–*FGFR1*; *BRAF* pV600E
Astrocytic gliomas	Pilocytic astrocytoma	Pilomyxoid astrocytoma; pilocytic astrocytoma with histological features of anaplasia	*KIA1549:BRAF* fusion; *NF1*; *BRAF* p.V600E; *FGFR1* mutation/fusion; *KRAS; RAF1* or *NTRK* fusion
	High-grade astrocytoma with piloid features		*NF1*; *FGFR*; *BRAF*:KIAA1549 fusion; often with homozygous deletion of *CDKN2A/B*
	Pleomorphic xanthoastrocytoma		*BRAF* pV600E typically with homozygous deletion of *CDKN2A/B*
	Subependymal giant cell astrocytoma		
	Astroblastoma		*MN1* fusion with *BEND2* or *CXXC5*
Glioneuronal/ neuronal tumors	Ganglioglioma		Most commonly *BRAF* p.V600E mutation, other MAPK pathway alterations
	Desmoplastic infantile ganglioglioma/astrocytoma		*BRAF* or *RAF1* fusions or mutations
	Dysembryoplastic neuroepithelial tumor		*FGFR1* mutation, fusion or intragenic duplication
	Diffuse glioneuronal tumor with oligodendroglioma-like features and nuclear clusters		Monosomy of chromosome 14
	Diffuse leptomeningeal glioneuronal tumor	With 1qgain; methylation class 1; methylation class 2	KIAA1549:BRAF fusion or other MAPK alteration, combined with 1p deletion
	Multinodular and vacuolating neuronal tumor		MAPK pathway
Ependymomas	Supratentorial ependymomas	ZFTA fusion-positive; YAP fusion-positive; additional molecular subgroups awaiting to be defined	*ZFTA* fusion most commonly with *RELA*; *YAP1* fusion most commonly with *MAMLD1*
	Posterior fossa ependymomas	Group A (PFA); group B (PFB)-retained H3K27 trimethylation; additional molecular subgroups awaiting to be defined	Loss of H3K27 trimethylation, EZHIP overexpression

(*) Adapted from [16].

**Table 2:** Medulloblastoma: molecular subgroups [24,29,30].

Imaging Characteristics	Wnt	SHH	Group 3	Group 4
Location	Cerebellar peduncle/cerebellopontine angle	Cerebellar hemispheres	Midline/fourth ventricle	Midline/fourth ventricle
Post-contrast enhancement	Variable	Present, intense	Present	Variable, can be non-enhancing
Drop metastasis	Rare	Rare	Frequent	Frequent
MRS	-	Prominent choline and lipids, low creatine, no or small taurine peak	Readily detectable taurine and creatine levels	Readily detectable taurine and creatine levels
